# Accuracy of Individual Rapid Tests for Serodiagnosis of *Gambiense* Sleeping Sickness in West Africa

**DOI:** 10.1371/journal.pntd.0003480

**Published:** 2015-02-02

**Authors:** Vincent Jamonneau, Oumou Camara, Hamidou Ilboudo, Moana Peylhard, Mathurin Koffi, Hassane Sakande, Louis N’Dri, Djénéba Sanou, Emilie Dama, Mamadou Camara, Veerle Lejon

**Affiliations:** 1 Centre International de Recherche-Développement sur l’Elevage en zones Subhumide (CIRDES), Bobo-Dioulasso, Burkina Faso; 2 Institut de Recherche pour le Développement (IRD), Unité Mixte de Recherche IRD-CIRAD 177, Montpellier, France; 3 Programme National de Lutte contre la Trypanosomose Humaine Africaine, Conakry, Guinea; 4 Université Jean Lorougnon GUEDE (UJLoG), UFR Environnement, Laboratoire des Interactions Hôte-Microorganismes-Environnement et Evolution (LIHME), Daloa, Côte d’Ivoire; 5 Institut Pierre Richet (IPR), Unité de Recherche « Trypanosomoses », Bouaké, Côte d’Ivoire; Institute of Tropical Medicine, BELGIUM

## Abstract

**Background:**

Individual rapid tests for serodiagnosis (RDT) of human African trypanosomiasis (HAT) are particularly suited for passive screening and surveillance. However, so far, no large scale evaluation of RDTs has been performed for diagnosis of *Trypanosoma brucei gambiense* HAT in West Africa. The objective of this study was to assess the diagnostic accuracy of 2 commercial HAT-RDTs on stored plasma samples from West Africa.

**Methodology/Principal findings:**

SD Bioline HAT and HAT Sero-K-Set were performed on 722 plasma samples originating from Guinea and Côte d’Ivoire, including 231 parasitologically confirmed HAT patients, 257 healthy controls, and 234 unconfirmed individuals whose blood tested antibody positive in the card agglutination test but negative by parasitological tests. Immune trypanolysis was performed as a reference test for trypanosome specific antibody presence. Sensitivities in HAT patients were respectively 99.6% for SD Bioline HAT, and 99.1% for HAT Sero-K-Set, specificities in healthy controls were respectively 87.9% and 88.3%. Considering combined positivity in both RDTs, increased the specificity significantly (p≤0.0003) to 93.4%, while 98.7% sensitivity was maintained. Specificities in controls were 98.7–99.6% for the combination of one or two RDTs with trypanolysis, maintaining a sensitivity of at least 98.1%.

**Conclusions/Significance:**

The observed specificity of the single RDTs was relatively low. Serial application of SD Bioline HAT and HAT Sero-K-Set might offer superior specificity compared to a single RDT, maintaining high sensitivity. The combination of one or two RDTs with trypanolysis seems promising for HAT surveillance.

## Author Summary

Screening for *gambiense* human African trypanosomiasis (HAT) or sleeping sickness is traditionally based on detection of trypanosome specific antibodies in blood. Whereas the card agglutination test is particularly suited for mass screening, individual rapid serodiagnostic tests (RDTs) are rather adapted for use in peripheral health-care centres. Two RDTs have been commercialized recently, and we assessed their diagnostic accuracy on stored plasma samples from West Africa. Immune trypanolysis was performed as a laboratory reference test for antibody presence. Although sensitivity for serodiagnosis of HAT in West Africa was high for both RDTs, their specificity was only 88%. Taking into account the high number of false positive test results, combined seropositivity in both RDTs was considered, raising specificity to 93%. Serial application of two RDTs should therefore be considered as an option for passive case finding, especially in settings with low HAT prevalence. A combination of one or two RDTs with immune trypanolysis further improved specificity for HAT to 99%, while maintaining sensitivity at 99% and seems promising for HAT surveillance.

## Introduction

Human African trypanosomiasis (HAT) or sleeping sickness is a fatal parasitic infection affecting rural populations in sub-Saharan Africa. During the last decade, active case finding by specialized mobile teams has considerably contributed to the reduction of the prevalence of HAT caused by *Trypanosoma brucei* (*T.b.*) *gambiense*. Since 2009, the number of cases reported annually has dropped below ten thousand. At low prevalence, cost-effectiveness of active screening decreases and passive case finding becomes increasingly important [1]. This shift from the mobile team to the fixed health system for HAT detection requires an adapted diagnostic approach. Detection of trypanosome specific antibodies in blood with the card agglutination test for trypanosomiasis (CATT), [2] is routinely applied for large scale active population screening. CATT is however ill-adapted to the conditions encountered in health-care centers. The limited shelf-life of the reconstituted CATT reagent at ambient temperature leads to considerable reagent loss when only few tests are performed per day. Another limitation of the CATT is the need of an agitator and a cold chain and therefore electric power, which are not always available in rural health-care centers.

The venue of individual rapid tests for serodiagnosis of HAT that are stable at ambient temperature and can be performed without additional material [3–5], is a key event in the development of an effective passive screening and HAT surveillance system [6]. Two rapid diagnostic tests (RDT) have been evaluated in phase 2 diagnostic trials [5, 7], show sufficient diagnostic accuracy and have been commercialized. So far, all RDT diagnostic evaluations have been performed on samples originating from Central-Africa, and no large scale evaluation has been performed for diagnosis of *gambiense* HAT in West Africa, while geographic variation in the accuracy of HAT serodiagnostic tests may occur [8].

The objective of this study was therefore to assess the diagnostic accuracy of 2 RDTs on stored plasma samples collected from HAT cases, negative controls, and serological suspects originating from Guinea and Côte d’Ivoire, two countries where HAT transmission is still active [9, 10].

## Materials and Methods

### Ethical statement

Samples were collected during medical surveys conducted by the national HAT control programs. All participants were informed about the study objectives in their own language and gave written informed consent. Children less than 12 years old were excluded. For participants between 12 and 18 years old, informed consent was obtained from the parents. Approval for this study was obtained from the consultative committee for deontology and ethics (Comité Consultatif de Déontologie et d’Ethique) of the Institut de Recherche pour le Développement. In Côte d’Ivoire, the protocol was approved by the national ethical committee (N°0308/MSLS/CNER-P).

### Origin of test samples

Plasma samples originated from subjects identified during active screening campaigns in the Dubreka, Boffa and Forecariah coastal mangrove HAT foci, situated north of Conakry in the Republic of Guinea and in the HAT foci of Oumé, Bouaflé, Sinfra, and Bonon in western central Côte d’Ivoire. All subjects underwent CATT/*T.b. gambiense* performed on whole blood (CATT-WB). Blood was collected in heparinised tubes and for CATT WB-positive persons, the plasma end titre was determined. All CATT-pl ≥1/4 positive persons underwent parasitological examination by direct microscopic examination of the lymph node aspirate if swollen lymph nodes were present and/or mini-anion exchange centrifugation technique on buffy coat (mAECT-BC) [11]. Based on the CATT and parasitological result, four categories of study participants (n = 722) were defined: 1° HAT: Parasitologically confirmed HAT patients with positive CATT-WB and CATT-pl end titer ≥1/4 (n = 229 from Guinea, n = 2 from Côte d’Ivoire); 2°Control: CATT-WB negative individuals for whom there was no suspicion for sleeping sickness infection (n = 101 from Guinea and n = 156 from Côte d’Ivoire); 3° SERO: Individuals with positive CATT-WB and CATT-pl end titer ≥1/4 (Seropositives) but no parasites detected (n = 123 from Guinea, n = 42 from Côte d’Ivoire); 4° SUSP: Individuals with positive CATT-WB but CATT-pl <1/4 (Suspects) in whom parasitological examinations were not performed (n = 69 from Côte d’Ivoire).

### RDT test procedure

Samples were retrospectively tested in two commercial RDTs for serodiagnosis of gambiense HAT: SD Bioline HAT (SD Diagnostics, Korea) and HAT Sero-*K*-Set (Coris BioConcept, Belgium). Both tests use purified native variant surface glycoproteins of *T.b. gambiense* variable antigen types LiTat 1.3 and 1.5 as antigens: SD Bioline HAT in two separate test lines (line 1 and 2 respectively), HAT Sero-*K*-Set in a single test line consisting of a mix of both glycoproteins.

The methodology applied was previously described for evaluation of RDTs for malaria diagnosis [12]. Tests were performed according to the indications of the manufacturers. Briefly, for SD Bioline HAT, 10 μls of test plasma were applied in the sample well, followed by 4 drops of assay diluent. For HAT Sero-*K*-Set, 15 μls of plasma were applied in the sample well, followed by 2 drops of BL-A buffer, after which the test device was re-inserted into its pouch. Tests were performed in batches of 10. Reading was done in day light, 15 minutes after application of the buffer. In case the control line did not appear, the test result was considered invalid. A scoring system was used for estimating the individual test line intensity: negative (no visible test line), faint (barely visible test line), weak (test line weaker than the control line), medium (test line equivalent to the control line) or strong (test line more intense than the control line) [13]. Reading was performed by 3 independent readers that were blind to other results (2 experienced and 1 less experienced that had been trained). The consensus test line intensity was based on consensus between two readers. In absence of consensus (3 different scores), the median score was taken. The test line was interpreted positive if the consensus test line intensity was faint or stronger. The HAT Sero-*K*-Set was positive if the test line was positive, the SD Bioline HAT was considered as positive if at least 1 test line was read as positive.

### Immune trypanolysis

For immune trypanolysis [14, 15], 25 μl of plasma were mixed with 25 μl of guinea pig serum and incubated for 30 minutes at room temperature. Blood of mice infected with *T.b. gambiense* was diluted in guinea pig serum to a final concentration of 10^7^ trypanosomes/ml. 50 μl of this trypanosome suspension were added. After 90 min of incubation at room temperature, the suspension was examined microscopically at 400x magnification. Trypanolysis was considered positive when 50–100% of the trypanosomes were lysed, otherwise it was considered negative. Two trypanolysis series were run, one with *T.b. gambiense* variable antigen type LiTat 1.3 and one with LiTat 1.5. A sample was considered positive in trypanolysis if it was positive with at least 1 variable antigen type.

### Analysis of results

Diagnostic sensitivity and specificity with binomial exact 95% confidence intervals (CI) were calculated for the results obtained in respectively the HAT and control group. Specificities and sensitivities were compared using the McNemar chi-square test. Differences between independent groups were assessed using a Chi squared test. Taking into account that the SERO and SUSP group are heterogeneous and might contain individuals that (i) are or have been in contact with *T.b. gambiense* but did not have detectable parasitemia, or (ii) are CATT false positives [16], immune trypanolysis was used as a reference test for presence of *T.b. gambiense* specific antibodies [15].

## Results

### Scoring of the RDT test line intensities; variability between readers

For both RDTs, not a single invalid RDT result was observed. The line intensities scored by the 3 readers as well as the consensus intensity are shown in Table 1. In HAT Sero-*K*-Set, the consensus test line intensity was negative for 370 persons, and faint to strong for 352 persons whom were considered positive. Absence of a consensus intensity or differences between individual scores larger than one grade occurred in 1.4% of readings (10/722). The kappa values for a positive or negative test result, when comparing each of the readers, were between 94.2 and 95.8%. The consensus test line intensity in SD Bioline HAT line 1 and 2 were respectively 362 and 363 times negative and 360 and 359 times positive. In respectively 3.8% (28/722) and 1.7% (12/722) of readings of line 1 and 2, at least one reader scored more than 1 grade different than another reader. At least 1 of both test lines scored positive in SD Bioline HAT for 396 persons. Kappas between readers were 91.7–95.0% for line 1, and 92.8–94.5% for line 2.

**Table 1:** Consensus intensity and intensity scores given by 3 readers to the test lines in HAT Sero-*K*-Set and SD Bioline HAT (line 1 and 2).

	**Score 2 readers**	**3^rd^ reader**					**Other intensity score combinations**
		**N**	**F**	**W**	**M**	**S**	
HAT Sero-*K*-Set consensus intensity							
N (n = 370)	N N	356	12		1	1	-
F (n = 14)	F F	8	1	4	0	0	N F W (n = 1)
W (n = 28)	W W	1	8	10	9	0	-
M (n = 96)	M M	2	0	14	44	35	W M S (n = 1)
S (n = 214)	S S	0	0	3	42	169	-
SD Bioline HAT line 1 consensus intensity							
N (n = 362)	N N	346	15	1	0	0	-
F (n = 45)	F F	16	18	11	0	0	-
W (n = 150)	W W	1	18	92	38	1	-
M (n = 72)	M M	0	0	22	10	18	W M S (n = 22)
S (n = 93)	S S	0	0	3	4	86	-
SD Bioline HAT line 2 consensus intensity							
N (n = 363)	N N	348	14	1	0	0	-
F (n = 47)	F F	15	21	10	0	0	N F W (n = 1)
W (n = 228)	W W	2	18	161	44	2	F W M (n = 1)
M (n = 65)	M M	1	0	34	17	9	W M S (n = 4)
S (n = 19)	S S	0	0	0	11	8	-

In case of a negative (N) consensus intensity the test line intensity was considered negative, the test line was interpreted positive if the consensus test line intensity was faint or stronger. N: negative, F: faint, W: weak, M: Medium, S: strong.

### Test result by participant category and diagnostic sensitivity and specificity

The number and proportion of positive test results by study participant category are summarized in Table 2. Sensitivities observed in HAT patients were respectively 99.6% (CI 97.6–100) for SD Bioline HAT, and 99.1% (CI 96.9–99.9) for HAT Sero-*K*-Set. There was no difference in sensitivity (p = 0.6) between the 2 RDTs. Specificities in healthy controls were respectively 87.9% (CI 83.3–91.7) for SD Bioline HAT and 88.3% (CI 83.8–92.0) for HAT Sero-*K*-Set. There was no difference in specificity between the lines 1 and 2 in SD Bioline HAT (p = 0.2), nor was there any difference in specificity between the 2 RDTs (p = 0.8). HAT Sero-*K*-Set was slightly more specific (p = 0.04) on samples from Côte d’Ivoire (91.7%, CI 86.2–95.5) than those from Guinea (83.2, CI 74.4–89.9), while no difference was observed with SD Bioline HAT (p = 0.7).

**Table 2:** Number of positive test results by type of study participant.

**Category**	**SD Bioline HAT**	**HAT Sero-*K*-Set**	**SD Bioline HAT + HAT Sero-*K*-Set**	**Immune trypanolysis**
HAT (n = 231)	230 (99.6%)	229 (99.1%)	228 (98.7%)	231 (100%)
Control (n = 257)	31 (12.1%)	30 (11.7%)	17 (6.6%)	11 (4.3%)
SERO (n = 165)	107 (64.8%)	84 (50.9%)	79 (47.9%)	77 (46.7%)
SUSP (n = 69)	28 (40.6%)	9 (13.0%)	7 (10.1%)	3 (4.3%)
**Total (n = 722)**	**396 (54.8%)**	**352 (48.8%)**	**331 (45.8%)**	**322 (44.6%)**

Considering combined positivity in both SD Bioline HAT and HAT Sero-*K*-Set, increased the specificity significantly to 93.4% (CI 89.6–96.1) compared to the single RDTs (p≤0.0003), while high sensitivity was maintained (p>0.16).

Sensitivity and specificity of immune trypanolysis were respectively 100% (CI 98.4–100) and 95.7% (CI 92.5–97.8). Immune trypanolysis was significantly more specific than SD Bioline HAT and HAT Sero-*K*-Set (p<0.0009). However, no significant difference in specificity could be observed between immune trypanolysis and the combination of SD Bioline HAT with HAT Sero-*K*-Set (p = 0.2).

In SD Bioline HAT, respectively 64.6% of SERO and 40.6% of SUSP tested positive. These percentages were respectively 50.9 and 13.0% for HAT Sero-*K*-Set, and respectively 47.9 and 10.1% for the combination of the 2 RDTs (Table 2). In immune trypanolysis, respectively 46.7 and 4.3% of SERO and SUSP were positive. Thus, significantly more SERO tested RDT or trypanolysis positive than SUSP (p≤0.001). With HAT Sero-*K*-Set or trypanolysis a similar proportion of SUSP and controls were positive (p≥0.1), while significantly more SUSP than controls tested positive in SD Bioline HAT (p<0.001).

### Presence of *T.b. gambiense* specific antibodies

Immune trypanolysis is considered to be the reference test for presence of trypanosome specific antibodies and *T.b. gambiense* contact. SD Bioline HAT and HAT Sero-*K*-Set were positive in respectively 93.5% (301/322, CI 90.2–95.9) and 94.4% (304/322, CI 91.3–96.7) of immune trypanolysis positive persons. In immune trypanolysis positives, there was no significant difference between both RDTs in number of positives (p = 0.4).

In immune trypanolysis negative persons, respectively 76.3% (305/400, CI 71.8–80.3) and 88.0% (352/400, CI 84.4–91.0) were negative in SD Bioline HAT and HAT Sero-*K*-Set. In this group, HAT Sero-*K*-Set was significantly more negative than SD Bioline HAT (p<0.0001).

Table 3 shows test line 1 and test line 2 results for SD Bioline HAT compared to trypanolysis with the corresponding variable antigen type, respectively LiTat 1.3 and LiTat 1.5. In SD Bioline HAT, line 1 and 2 were significantly more positive than the corresponding variable antigen type in immune trypanolysis (p≤0.0001). Among the samples that were trypanolysis negative for both LiTat 1.3 and LiTat 1.5, there was no significant difference in test line 1 or 2 positivity, nor was there in trypanolysis positive samples (p values of 0.2).

**Table 3:** Reactivity of individual test lines in SD Bioline HAT in function of trypanolysis results of the corresponding *T.b. gambiense* variable antigen type.

	**TL LiTat 1.3**			**TL LiTat 1.5**	
**SD Bioline HAT**	**Negative**	**Positive**	**SD Bioline HAT**	**Negative**	**Positive**
Line 1 negative	326	36	Line 2 negative	343	20
Line 1 positive	78	282	Line 2 positive	73	286

### RDT combined with immune trypanolysis, by participant category

Table 4 shows the number of positives in one or both RDTs combined with trypanolysis, considering only those subjects positive that are positive in all individual tests. Sensitivities in HAT patients were respectively 99.6% (CI 97.6–100) for the combination SD Bioline HAT and trypanolysis, 99.1% (CI 96.9–99.9) for HAT Sero-*K*-Set combined with trypanolysis, and 98.7% (CI 96.3–99.7) for the combination of the 2 RDTs with trypanolysis. There was no difference in sensitivity between the 3 different test combinations (p>0.2). Specificities in controls were respectively 98.8% (CI 96.6–99.8) for the combination SD Bioline HAT and trypanolysis, 98.1% (CI 95.5–99.4) for HAT Sero-*K*-Set combined with trypanolysis, and 99.2% (CI 97.2–99.9) for the combination of the 2 RDTs with trypanolysis. No significant differences were observed between the specificities of the different test combinations (p>0.08). However, the combination of one or 2 RDTs with immune trypanolysis was more specific than one or 2 RDTs without immune trypanolysis (p<0.005).

**Table 4:** Number of positive test results combining RDTs with trypanolysis, by type of study participant.

**Category**	**SD Bioline HAT + TL**	**HAT Sero-*K*-Set + TL**	**SD Bioline HAT + HAT Sero-*K*-Set + TL**
HAT (n = 231)	230 (99.6%)	229 (99.1%)	228 (98.7%)
Control (n = 257)	3 (1.2%)	5 (1.9%)	2 (0.8%)
SERO (n = 165)	66 (40.0%)	68 (41.2%)	63 (38.2%)
SUSP (n = 69)	2 (2.9%)	2 (2.9%)	2 (2.9%)
**Total (n = 722)**	**301 (41.9%)**	**304 (42.1%)**	**295 (40.9%)**

The combination of one or two RDTs with trypanolysis was positive in 38.2–41.2% of SERO, 2.9% of SUSP (Table 4). Again, significantly more SERO tested positive than SUSP (p≤0.001), while a similar proportion of SUSP and controls were positive (p≥0.6).

## Discussion

This is the first study to report on HAT diagnostic accuracy on a large number of samples originating from West Africa, and also the first to perform both commercially available RDTs for serodiagnosis of HAT on the same sample set. Although sensitivity of the two tested RDTs for serodiagnosis of HAT in West Africa was high, specificity remained limited to 88%. Specificity significantly increased to 93% considering combined seropositivity in both RDTs. Using a combination of one or two RDTs with trypanolysis further improved specificity to 99% while maintaining sensitivity at 99%.

For interpretation of the results, a selection bias caused by routine screening of the population at risk using the CATT test should be taken into account. This could result in an overestimation of test sensitivity and specificity, as CATT consists of whole fixed and stained trypanosomes of the LiTat 1.3 variable antigen type and the corresponding purified native VSG is one of the two antigens used in both RDTs as well. Furthermore the evaluation was done on stored plasma samples and not on fresh whole blood. We cannot exclude that this could influence the test results, although antibodies are well conserved after freezing. Subjectivity of scoring of the RDT test result was largely eliminated by the use of 3 independent readers. Absence of a consensus intensity or the occurrence of large differences between scores, were not frequent but can be explained by a non-uniform coloration of the test line.

The RDT specificities around 88% observed in this study are close to the 87% specificity mentioned in the SD Bioline HAT test instructions (version 53FK10–04-En-0) but below the previously observed specificities of 98.6% for HAT-Sero-*K*-Set [7] and of 94.6% for a SD Bioline HAT prototype [5]. Specificity of both RDTs was also below the 98.7% specificity of CATT on whole blood previously reported in West Africa [15]. Possible explanations could be regional differences [8], cross reaction with other infections or superior challenge by animal trypanosomes to cause false positive reactions [15], or other.

Although immune trypanolysis has been considered 100% specific for HAT [15], 4.3% of controls tested positive. It is not clear if this is due to false positivity, if previously treated HAT cases who did not declare themselves were included as controls, or if they were trypanotolerant individuals who became negative in CATT but remained immune trypanolysis positive [17]. The phenomenon of immune trypanolysis positive, CATT negative healthy controls requires further examination.

Taking into account the high number of false positive test results observed, we examined the possible performance of combined positivity in both RDTs for diagnosis of HAT, taking the example of the strategy of serial testing applied with RDTs for diagnosis of HIV [18]. Although both RDTs actually available for serodiagnosis of HAT are based on identical antigens, considering combined positivity significantly increased specificity and reduced the number of false positives by almost half. Serial application of SD Bioline HAT and HAT Sero-*K*-Set could therefore be considered as an option for passive case finding, as long as no second generation RDTs for serodiagnosis of HAT are available based on different antigens. However, as the combined specificity of 93.4% is still suboptimal, the local context, on-site availability of parasitological confirmation tests and the relative cost should be taken into account when deciding on test algorithms.

For surveillance of HAT, RDTs are actually being implemented in fixed health centres. In case of clinical suspicion and a positive RDT, and depending on the experience of the health centres in HAT diagnosis, local prevalence, and availability of sensitive confirmation diagnostic tests, blood on filter paper is sampled and sent to a reference centre for immune trypanolysis, either directly, or after unsuccessful parasitological examination. Those persons with a trypanolysis positive result are considered at high suspicion for infection, should be (re-)examined parasitologically and followed-up closely. Although in this study stored plasma samples were used for immune trypanolysis instead of filter paper, our results show the potential high diagnostic accuracy of a combined RDT-trypanolysis approach. In the final result no difference in accuracy occurred when combining one or two RDTs followed by trypanolysis. However, the serial application of two RDTs may present considerable advantages. The number of unnecessary parasitological examinations may be significantly reduced as well as the number of filter papers to be dispatched and tested in trypanolysis. Use of filter paper instead of plasma for immune trypanolysis, may further decrease of the number of trypanolysis positive SERO and SUSP individuals [19] thus further decrease the number of people to be followed up.

Our data suggest that the specificity of actual RDTs for serodiagnosis of HAT might be lower than expected. Care should therefore be taken in interpretation of the result, especially since the future use of RDTs alone, without parasitological confirmation, for patient management has already been suggested [20]. Serological screening using serial application of SD Bioline HAT and HAT Sero-K-Set might offer superior specificity compared to a single RDT, maintaining high sensitivity. The combination of one or two RDTs with trypanolysis seems promising for HAT surveillance. However, the diagnostic accuracy and especially the specificity of applying a combination of RDTs on fresh blood for HAT diagnosis, without prior CATT selection, remains to be determined as well as their combination with trypanolysis on filter paper, not only in West Africa but also in Central Africa.

## Supporting Information

S1 Checklist STARD checklist. (DOCX)
